# The clinical significance of splice variants and subcellular localisation of survivin in non-small cell lung cancers

**DOI:** 10.1038/sj.bjc.6604253

**Published:** 2008-02-19

**Authors:** J Nakano, C Huang, D Liu, D Masuya, H Yokomise, M Ueno, R Haba, S Sumitomo

**Affiliations:** 1 Second Department of Surgery, Faculty of Medicine, Kagawa University, 1750-1, Ikenobe, Miki-cho, Kita-gun, Kagawa 761-0793, Japan; 2 Department of Pathology and Host Defense, Kagawa University, Kagawa, Japan; 3 Department of Diagnostic Pathology, Kagawa University, Kagawa, Japan; 4 Department of Thoracic Surgery, Japanese Red Cross Society Wakayama Medical Center, 20 Komatsubara-douri, Wakayama-shi, Wakayama 640-8558, Japan

**Keywords:** survivin, survivin-deltaEx3, nuclear survivin, proliferation, apoptosis, lung cancer

## Abstract

Survivin is a member of the inhibitor of apoptosis protein family. Survivin has splice variants with different biological functions associated with tumorigenesis. We investigated 134 non-small cell lung cancers (NSCLCs) to study the clinical significance of wild-type *survivin*, *survivin-2B*, and *survivin-deltaEx3*. Real-time PCR analyses were performed for their gene expressions. The subcellular localisation of survivin proteins was evaluated by immunohistochemistry. The Ki-67 proliferation index and the apoptotic index were also evaluated. The *survivin-deltaEx3* gene expression was significantly higher in stage II–III than in stage I (*P*=0.0174), and significantly correlated with the nuclear pan-survivin expression (*P*<0.0001). The Ki-67 index was significantly higher in wild-type *survivin*-positive tumours (*P*<0.0001), *survivin-deltaEx3*-positive tumours (*P*<0.0001), and tumours with positive expression of the nuclear pan-survivin (*P*=0.0047). In contrast, the apoptotic index was significantly lower only in wild-type *survivin*-positive tumours (*P*<0.0001). Thus, the wild-type *survivin* gene expression was associated with apoptotic inhibition and tumour proliferation. Furthermore, the *survivin-deltaEx3* gene expression was strongly associated with tumour proliferation, especially in advanced stage NSCLCs. In contrast, the *survivin-2B* gene expression did not correlate with tumour proliferation or tumour apoptosis.

Non-small cell lung cancer (NSCLC) has become the leading cause of cancer death in Japan. Clarifying the mechanism of tumour biology is considered to be important for improving the clinical outcome of advanced NSCLC patients. Owing to recent developments in molecular biology, many molecular markers have proven to be associated with cell cycle regulation, apoptosis, and chemo-radio resistance. These molecular markers include survivin (Altieri, 2003), p53 (Vogelstein and Kinzler, 1992), and bcl-2 (Strasser *et al*, 1997).

Survivin was originally identified as a member of the inhibitor of apoptosis protein (IAP) family (Ambrosini *et al*, 1997). To date, survivin has at least five splice variants; including wild-type survivin (i.e. survivin itself), survivin-2*α*, survivin-2B, survivin-deltaEx3, and survivin-3B (Mahotka *et al*, 1999; Badran *et al*, 2004; Caldas *et al*, 2005a). Intriguingly, these splice variants are reported to have different biological functions (Mahotka *et al*, 2002a). Among them, recent studies found both wild-type survivin and survivin-deltaEx3 to demonstrate not only apoptotic inhibition but also an acceleration of cell proliferation (Honda *et al*, 2003; Song and Wu, 2005; Takashima *et al*, 2005). In contrast, survivin-2B is considered to attenuate the antiapoptotic function of wild-type survivin (Caldas *et al*, 2005b).

The ability to control tumour proliferation and apoptosis may enable us to develop new strategies for the treatments of NSCLC patients. Therefore, we performed a study on the gene expressions of wild-type *survivin*, *survivin-2B*, and *survivin-deltaEx3* in relation to the subcellular localisation of survivin protein, the Ki-67 proliferation index (Gerdes *et al*, 1984), and the apoptotic index (Tanaka *et al*, 2001) to clarify the clinical significance of these expressions in NSCLCs.

## MATERIALS AND METHODS

### Clinical characteristics of patients

From June 1999 to December 2002, NSCLC patients, who underwent surgery at the Second Department of Surgery, Kagawa University or the Department of Thoracic Surgery, Japanese Red Cross Society Wakayama Medical Center, were studied. This study was approved by the institutional review board of Kagawa University (14-7, a clinical study of biological markers in NSCLCs). Signed, written informed consent was obtained from all patients before therapy was initiated. In total, 134 patients up to stage IIIB were investigated. The patients' clinical records and histopathological diagnoses were fully documented.

### Real-time PCR for gene expressions

The total cellular RNA was extracted from frozen tissue specimens by the acid guanidinium thiocyanate procedure. First-strand cDNA synthesis was performed with 5 *μ*g of total RNA using a cDNA synthesis kit (Pharmacia, Piscataway, NJ, USA). Because real-time PCR (RT–PCR) is not an appropriate method to discriminate between wild-type *survivin* and other splice variants, we carried out quantitative RT–PCR assays using densitometric analyses of gel electrophoresis to evaluate the gene expressions of wild-type *survivin*, *survivin-2B,* and *survivin-deltaEx3.* For the gene expressions of wild-type *survivin*, *survivin-2B*, and *survivin-deltaEx3*, oligonucleotides were synthesised as PCR primers based on the published reports (Yamada *et al*, 2003). The nucleotide sequence 5′-CCACCGCATCTCTACATTCA-3′ was used as the sense primer of wild-type *survivin* and *survivin-deltaEx3*, 5′-GAGGCTGGCTTCATCCACTG-3′ was used as the sense primer of *survivin-2B*, 5′-TATGTTCCTCTATGGGGTCG-3′ as the antisense primer of wild-type *survivin*, 5′-GTTCCTCTCTCGTGATCCG-3′ as the antisense primer of *survivin-2B*, and 5′-TTTCCTTTGCATGGGGTC-3′ as the antisense primer of *survivin-deltaEx3.* All subsequent assays were carried out using the parameters that yielded amplification of wild-type *survivin*, *survivin-2B, survivin-deltaEx3*, and *β*-*actin* DNA (the internal control) within a linear range. The reaction mixture of wild-type *survivin or survivin-2B* was subjected to 36 PCR amplification cycles of 60 s at 94°C, 60 s at 60°C, and 90 s at 72°C, and that of *survivin-deltaEx3* was subjected to 36 PCR amplification cycles of 60 s at 94°C, 60 s at 55°C, and 90 s at 72°C, and that of *β-actin* was subjected to 31 PCR amplification cycles of 60 s at 94°C, 60 s at 60°C, and 90 s at 72°C. Preparations of a human adenocarcinoma cell line, A549, were used as positive controls for gene expressions of wild-type *survivin*, *survivin-2B*, and *survivin-deltaEx3*. The amplified DNA samples were run on a 1% agarose gel, and the bands were visualised with ethidium bromide. The densitometric value obtained for a wild-type *survivin*, *survivin-2B*, and *survivin-deltaEx3* band in a given tumour sample was divided by the value of the *β*-*actin*, and the resultant ratio was referred to as the gene expression ratio. Thereafter, the expression ratio for a given tumour sample was divided by the expression ratio of A549 to obtain the standardised gene expression ratio.

### Immunohistochemistry

We used a mouse monoclonal antibody against pan-survivin (sc17779; Santa Cruz Biotechnology Inc., Santa Cruz, CA, USA) diluted at 1 : 50, and a mouse monoclonal antibody for the Ki-67 antigen (MIB-1; DAKO, Glostrup, Denmark) diluted at 1 : 40. Formalin-fixed paraffin-embedded tissue was cut into 4-*μ*m sections and mounted on poly-L-lysine-coated slides. Sections were deparaffinised and rehydrated. The slides were then heated in a microwave for 10 min in a 10 *μ*mol l^−1^ citrate buffer solution at pH 6.0. After quenching the endogenous peroxidase activity with 0.3% H_2_O_2_ (in absolute methanol) for 30 min, the sections were treated for 2 h with 5% bovine serum albumin to block nonspecific staining. Duplicated sections were incubated overnight with primary antibodies, respectively. Slides were then incubated for 1 h with biotinylated secondary antibodies (Vector Laboratories Inc., Burlingame, CA, USA). The sections were incubated with the avidin–biotin–peroxidase complex (Vector) for 1 h, and antibody binding was visualised with 3,3′-diaminobenzidine tetrahydrochloride. Finally, the sections were lightly counterstained with Mayer's haematoxylin.

All immunostained sections were independently evaluated by two authors (JN and MU), without knowledge of the patients' characteristics. At least 200 tumour cells were scored per × 40 field. Regarding pan-survivin expression, the nuclear staining and the cytoplasmic staining in each section were evaluated, respectively, in a semiquantitative manner that reflected both the intensity and percentage of cells staining at each intensity. The intensity was classified as 0 (no staining), +1 (weak staining), +2 (distinct staining), or +3 (very strong staining). A value designated as the ‘HSCORE’ thus was obtained for each slide by using the following algorithm: HSCORE=Σ (*I* × PC), where *I* and PC represent the intensity and the percentage of cells that stain at each intensity, respectively. The percentage of carcinoma cells with positive staining for Ki-67 antigen was scored as the Ki-67 proliferation index.

### Detection of apoptosis

The presence of apoptotic cells was detected with the TUNEL method using the *In Situ* Apoptosis Detection Kit (Takara Biomedicals, Otsu, Japan). After sections were deparaffinised and rehydrated, the slides were treated for 15 min with 20 *μ*g ml^−1^ Proteinase K. After quenching the endogenous peroxidase activity with 3% H_2_O_2_ for 5 min, the sections were then incubated for 90 min at 37°C with the TUNEL reaction mixture, including terminal deoxynucleotidyl transferase (TdT). Next, the sections were incubated for 30 min at 37°C with anti-FITC horseradish peroxidase conjugate. Staining was developed using 3,3′-diaminobenzidine tetrahydrochloride for 15 min. Finally, the sections were lightly counterstained with Mayer's haematoxylin. Sections incubated with the TUNEL reaction mixture without TdT were used as negative control slides. Apoptotic cells were determined based on observations of TUNEL-staining sections and serial HE-staining sections. Cells staining positive for TUNEL, if they represented the histologic features of necrosis in HE-staining sections, were not considered to be apoptotic cells. In each case, a total of 10 000 tumour cells were evaluated at high magnification by two authors (JN and MU) independently, without knowledge of the patients' characteristics. The apoptotic index was defined as the number of apoptotic cells per 1000 tumour cells.

### Statistical analysis

The ratio of the Ki-67 proliferation index divided by the apoptotic index was referred to as the growth index for each sample (Tsoli *et al*, 2002). The statistical significances of wild-type *survivin*, *survivin-2B*, and *survivin-deltaEx3* gene expression in relation to several clinical and pathological parameters were assessed by *t*-test or an analysis of variance with Bonferroni/Dunn test. Because the wild-type *survivin* expression cutoff line of 1.0 demonstrated the most significance in relation to the Ki-67 proliferation index and the apoptotic index, the sample was classified as a wild-type *survivin*-positive tumour when the standardised wild-type *survivin* gene expression was >1.0. Because the *survivin-deltaEx3* expression cutoff line of 0.5 demonstrated the most significance in relation to the Ki-67 proliferation index, the sample was classified as a *survivin-deltaEx3*-positive tumour when the standardised *survivin-deltaEx3* gene expression was >0.5. The sample was classified as a *survivin-2B*-positive tumour when the standardised *survivin-2B* expression was >1.0 (a median value of *survivin-2B* gene expression). Samples were considered to have a positive expression of nuclear pan-survivin if the HSCORE of the nuclear staining of pan-survivin was more than 10, a positive expression of cytoplasmic pan-survivin if the HSCORE of the cytoplasmic staining of pan-survivin was more than 50. All *P*-values were based on two-tailed statistical analysis and a *P*-value of <0.05 was considered to indicate statistical significance.

## RESULTS

### Wild-type *survivin* gene expression in NSCLCs

**Figure 1:**
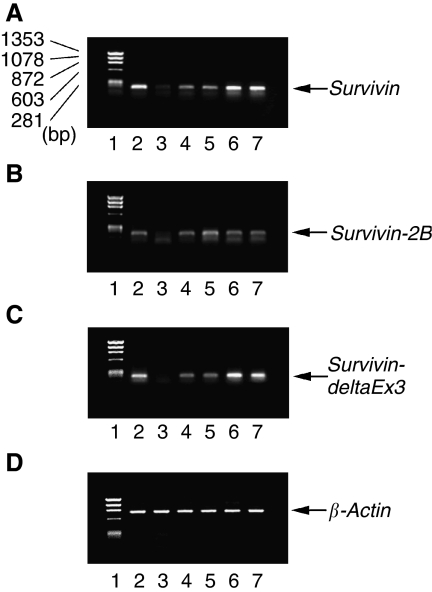
Agarose gel electrophoresis of RT–PCR-amplified (**A**) wild-type survivin cDNA, (**B**) survivin-2B cDNA, (**C**) survivin-deltaEx3 cDNA, and (**D**) *β*-actin cDNA (internal PCR control). Lane 1, size marker; lane 2, A549 (positive control); lane 3, normal lung tissue; lanes 4 and 5, low expression of both wild-type *survivin* and *survivin-deltaEx3*; lanes 6 and 7, high expression of both wild-type *survivin* and *survivin-deltaEx3*.RT–PCR=real-time PCR.

**Figure 2:**
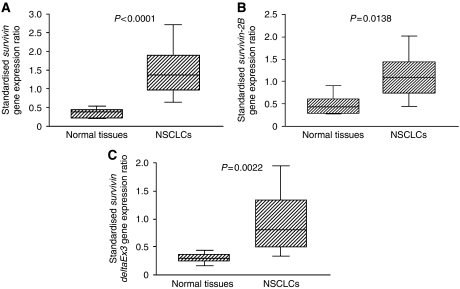
Standardised gene expression ratio in normal lung tissues and tumour tissues. (**A**) Wild-type *survivin*, (**B**) *survivin-2B*, and (**C**) *survivin-deltaEx3*.

We studied the wild-type *survivin* gene expression in eight noncancerous samples to evaluate its expression in normal lung tissue. The standardised wild-type *survivin* gene expression ratio was low in normal lung tissue specimens (mean 0.363±0.135). In contrast, the standardised *survivin* gene expression ratio varied greatly among the 134 tumour tissue specimens (mean 1.531±0.795, Figure 1A). The standardised wild-type *survivin* gene expression ratio was significantly higher in tumour tissue specimens than in normal lung tissue specimens (*P*<0.0001, Figure 2A). Seventy-two carcinomas (53.7%) were wild-type *survivin*-positive tumours. Regarding tumour histology, the wild-type *survivin* gene expression was significantly higher in squamous cell carcinomas than in adenocarcinomas (1.690±0.892 *vs* 1.362±0.664, *P*=0.0181, Table 1). In addition, the wild-type *survivin* gene expression was significantly higher in moderately or poorly differentiated tumours than in well-differentiated tumours (1.614±0.807 *vs* 1.288±0.712, *P*=0.0383).

### *Survivin-2B* gene expression in NSCLCs

The standardised *survivin-2B* gene expression ratio was low in eight normal lung tissue specimens (mean 0.496±0.246). In contrast, the standardised *survivin-2B* gene expression ratio also varied greatly among the tumour tissue specimens (mean 1.233±0.830, Figure 1B). The standardised *survivin-2B* gene expression ratio was significantly higher in tumour tissues than in normal lung tissues (*P*=0.0138, Figure 2B). However, no difference was observed in the *survivin-2B* gene expression regarding tumour histology, tumour differentiation, or any other clinicopathological parameter (Table 1).

### *Survivin-deltaEx3* gene expression in NSCLCs

The standardised *survivin-deltaEx3* gene expression ratio was low in eight normal lung tissue specimens (mean 0.296±0.098). In contrast, the standardised *survivin-deltaEx3* gene expression ratio varied greatly among the tumour tissue specimens (mean 0.971±0.610, Figure 1C). The standardised *survivin-deltaEx3* gene expression ratio was significantly higher in tumour tissues than in normal lung tissues (*P*=0.0022, Figure 2C). One hundred and one carcinomas (75.4%) were *survivin-deltaEx3-*positive tumours. Regarding tumour histology, the *survivin-deltaEx3* gene expression was also significantly higher in squamous cell carcinomas than in adenocarcinomas (1.075±0.616 *vs* 0.854±0.587, *P*=0.0377, Table 1). Regarding tumour differentiation, the *survivin-deltaEx3* gene expression was significantly higher in moderately or poorly differentiated tumours than in well-differentiated tumours (1.049±0.619 *vs* 0.743±0.526, *P*=0.0110). Regarding the clinicopathological characteristics, the *survivin-deltaEx3* gene expression was significantly higher in stage II–III NSCLCs than in stage I NSCLCs (1.084±0.661 *vs* 0.833±0.512, *P*=0.0174).

**Figure 3:**
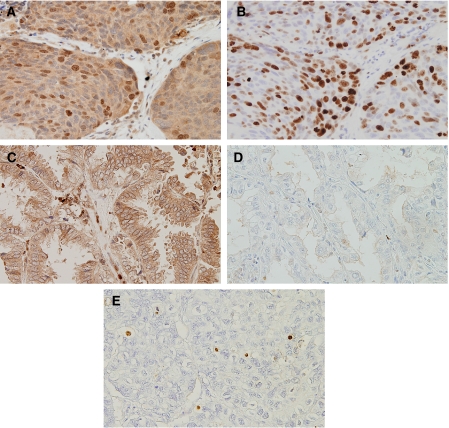
Immunostaining of lung cancers. Squamous cell carcinoma with (**A**) strongly positive expression of both nuclear and cytoplasmic pan-survivin, (**B**) high Ki-67 index. Adenocarcinoma with only (**C**) positive expression of cytoplasmic pan-survivin, (**D**) low Ki-67 index. (**E**) Adenocarcinoma with high apoptotic index.

Subcellular localisation of the survivin protein expression in relation to wild-type *survivin*, *survivin-2B*, and *survivin-deltaEx*3 gene expressions. Immunostaining using the antibody against pan-survivin showed various patterns of cytoplasmic staining and nuclear staining (Figure 3A and C). Regarding the cytoplasmic staining, 98 carcinomas (73.1%) had a positive expression of cytoplasmic pan-survivin. However, the HSCORE of the cytoplasmic pan-survivin did not correlate with the gene expression of wild-type *survivin, survivin-2B*, *or survivin-deltaEx3*.

On the other hand, regarding the nuclear staining, 88 carcinomas (65.7%) had a positive expression of nuclear pan-survivin. Furthermore, the standardised *survivin-deltaEx3* gene expression significantly correlated with the HSCORE for the nuclear staining of pan-survivin (*r*=0.642, *P*<0.0001). However, the HSCORE of the nuclear pan-survivin did not correlate with the gene expression of wild-type *survivin or survivin-2B*.

### Ki-67 proliferation index in NSCLCs

**Figure 4:**
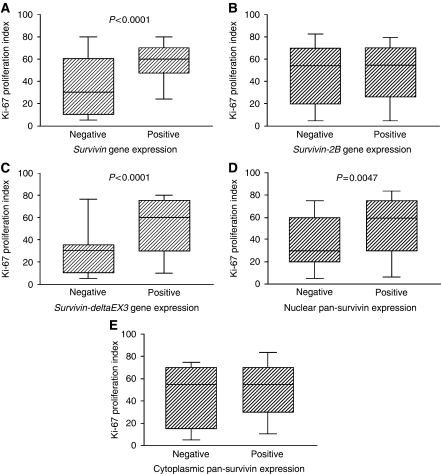
Ki-67 proliferation index in relation to wild-type *survivin* gene expression (**A**), *survivin-2B* gene expression (**B**), *survivin-deltaEx3* gene expression (**C**), nuclear pan-survivin expression (**D**), and cytoplasmic pan-survivin expression (**E**).

The Ki-67 proliferation index ranged from 2.0 to 95.0 (mean 47.6±27.3, Figure 3B and D). Regarding the gene expressions of splice variants of survivin, the Ki-67 proliferation index was significantly higher in the wild-type *survivin-*positive tumours than in the wild-type *survivin*-negative tumours (57.4±22.0 *vs* 36.2±28.5, *P*<0.0001, Figure 4A, Table 2). However, there was no difference in the Ki-67 proliferation index according to the *survivin-2B* gene expression (Figure 4B). In contrast, the Ki-67 proliferation index was also significantly higher in the *survivin-deltaEx3-*positive tumours than in the *survivin-deltaEx3*-negative tumours (53.4±25.5 *vs* 29.8±25.0, *P*<0.0001, Figure 4C). Regarding the subcellular localisation of pan-survivin protein, the Ki-67 proliferation index was significantly higher in tumours with a positive expression of nuclear pan-survivin than in tumours with a negative expression of nuclear pan-survivin (52.4±26.5 *vs* 38.5±26.6, *P*=0.0047, Figure 4D). In contrast, there was no difference in the Ki-67 proliferation index according to the cytoplasmic pan-survivin expression (Figure 4E).

### Apoptotic index in NSCLCs

**Figure 5:**
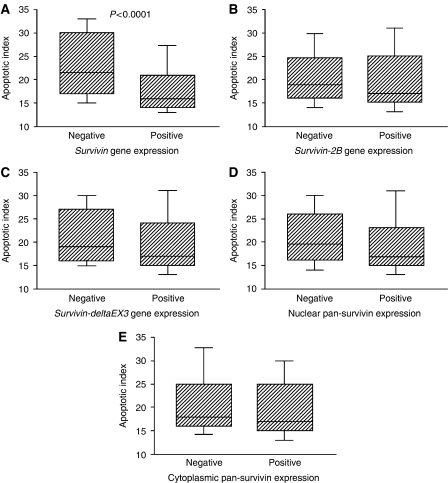
Apoptotic index in relation to wild-type *survivin* gene expression (**A**), *survivin-2B* gene expression (**B**), *survivin-deltaEx3* gene expression (**C**), nuclear pan-survivin expression (**D**), and cytoplasmic pan-survivin expression (**E**).

The apoptotic index ranged from 10.0 to 40.0 (mean 20.7±7.4, Figure 3E). Regarding the gene expressions of splice variants of survivin, the apoptotic index was significantly lower in the wild-type *survivin-*positive tumours than in the wild-type *survivin*-negative tumours (18.1±5.6 *vs* 23.1±7.2, *P*<0.0001, Figure 5A). On the other hand, there was no difference in the apoptotic index according to the *survivin-2B* expression or *survivin-deltaEx3* expression (Figure 5B and C). Regarding the pan-survivin protein expression, neither the nuclear pan-survivin expression nor the cytoplasmic pan-survivin expression was associated with the apoptotic index (Figure 5D and E).

### Growth index in NSCLCs

**Figure 6:**
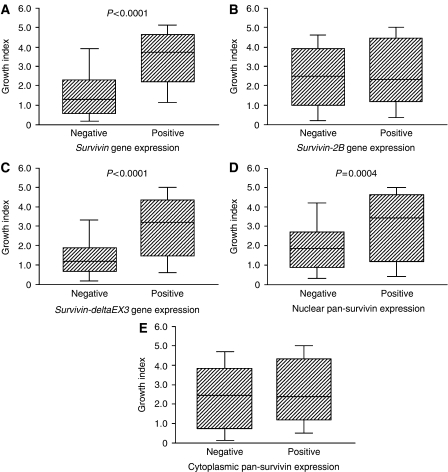
Growth index in relation to wild-type *survivin* gene expression (**A**), *survivin-2B* gene expression (**B**), *survivin-deltaEx3* gene expression (**C**), nuclear pan-survivin expression (**D**), and cytoplasmic pan-survivin expression (**E**).

The growth index ranged from 0.05 to 9.00 (mean 2.62±1.79). The growth index was significantly higher in the wild-type *survivin-*positive tumours than in the wild-type *survivin*-negative tumours (3.47±1.68 *vs* 1.66±1.37, *P*<0.0001, Figure 6A). However, there was no difference in the growth index according to the *survivin-2B* gene expression (Figure 6B). In contrast, the growth index was also significantly higher in the *survivin-deltaEx3-*positive tumours than in the *survivin-deltaEx3*-negative tumours (3.01±1.76 *vs* 1.48±1.33, *P*<0.0001, Figure 6C). Regarding the subcellular localisation of pan-survivin protein, the growth index was significantly higher in tumours with a positive expression of nuclear pan-survivin than in tumours with a negative expression of nuclear pan-survivin (3.02±1.88 *vs* 1.90±1.33, *P*=0.0004, Figure 6D). In contrast, there was no difference in the growth index according to the cytoplasmic pan-survivin expression (Figure 6E).

## DISCUSSION

Survivin is reported to be expressed in most human malignancies (Kawasaki *et al*, 1998; Monzo *et al*, 1999; Sui *et al*, 2002), while its expression is absent in normal differentiated tissues (Salvesen and Duckett, 2002). Although survivin was initially identified as a member of the IAP family (Ambrosini *et al*, 1997), recent studies have demonstrated that survivin not only inhibits the caspase-dependent apoptotic pathway, but also accelerates cell proliferation (Honda *et al*, 2003).

Survivin is known to have at least five spliced variants. These splice variants have different biological functions (Mahotka *et al*, 2002a). Among them, recent experimental studies demonstrated that survivin-deltaEx3 also inhibits apoptosis and induces cell proliferation, similar to wild-type survivin (Song and Wu, 2005; Takashima *et al*, 2005). In contrast, survivin-2*α* and survivin-2B are considered to attenuate the antiapoptotic function of wild-type survivin (Caldas *et al*, 2005b).

As a result, these splice variants are variably involved in the biological functions associated with carcinogenesis, including cell proliferation, apoptosis, and chemo-radio resistance. Previous clinical studies have reported the survivin expression to be associated with tumour cell proliferation, apoptotic inhibition, and a poor prognosis in various human cancers, including NSCLCs (Martinez *et al*, 2004; Shinohara *et al*, 2005). However, only a few clinical reports studied these splice variants of survivin in human cancers (Mahotka *et al*, 2002a; Meng *et al*, 2004; Ling *et al*, 2005; Taubert *et al*, 2005). Therefore, to clarify the clinical significance of these splice variants of survivin in NSCLCs, we performed the present study on the gene expressions of wild-type *survivin*, *survivin-2B*, and *survivin-deltaEx3* in relation to the subcellular localisation of survivin proteins, tumour cell proliferation, and apoptosis.

In the present study, regarding the relationship of the expression of these splice variants of *survivin*, the *survivin-deltaEx3* gene expression significantly correlated with the wild-type *survivin* gene expression (*r*=0.646, *P*<0.001). However, the correlation between the *survivin-2B* gene expression and the wild-type *survivin* gene expression was low (*r*=0.254). Regarding the biological significance of these splice variants of *survivin*, the present study also demonstrated the wild-type *survivin* gene expression to be associated with both tumour cell proliferation and apoptotic inhibition. In contrast, the *survivin-deltaEx3* gene expression correlated with the nuclear pan-survivin expression. Furthermore, both the *survivin-deltaEx3* gene expression and the nuclear pan-survivin expression were found to be associated with tumour cell proliferation, but not with apoptotic inhibition. However, the *survivin-2B* gene expression did not correlate with tumour cell proliferation or apoptotic inhibition. In total, the growth index was associated with both the wild-type *survivin* expression and the *survivin-deltaEx3* expression. As a result, the present study revealed wild-type *survivin* to be associated with both tumour cell proliferation and apoptotic inhibition, while *survivin-deltaEx3* was strongly associated with tumour cell proliferation.

In fact, previous clinical studies also revealed the survivin-deltaEx3 expression to be associated with a high proliferation rate (Takashima *et al*, 2005). Recent studies have revealed that the *survivin-deltaEx3* overexpression is a significant poor prognostic factor in cancer patients, including NSCLCs (Ling *et al*, 2005; Taubert *et al*, 2005). Furthermore, the present study found the *survivin-deltaEx3* gene expression to be significantly higher in advanced-stage NSCLCs. Therefore, the *survivin-deltaEx3* gene overexpression could produce more aggressive tumours with a high proliferation rate in advanced-stage NSCLCs. Because the biomarkers associated with tumour proliferation are significant factors in advanced-stage NSCLCs (Huang *et al*, 2005), the level of the *survivin-deltaEx3* gene expression could thus affect the clinical impact of NSCLCs, especially in the advanced stages. A recent study on soft tissue sarcoma also reported the overexpression of *survivin-deltaEx3* gene to correlate with an advanced clinical stage and a poor prognosis (Taubert *et al*, 2005).

On the other hand, previous immunohistochemical studies revealed the nuclear expression of survivin proteins also to be associated with tumour cell proliferation and a poor prognosis in cancer patients, as was also shown in the present study, while the cytoplasmic expression of survivin proteins was not (Martinez *et al*, 2004; Huang *et al*, 2005; Ling *et al*, 2005; Shinohara *et al*, 2005; Taubert *et al*, 2005). These studies were performed using antibodies against pan-survivin, which cannot distinguish wild-type survivin from other spliced variants. In general, wild-type survivin is considered to be located both in the cytoplasm for apoptotic inhibition, and in the nuclei for cell proliferation. In contrast, survivin-deltaEx3 is probably located in the nucleus because of its nuclear localisation signal (Mahotka *et al*, 2002b; Song and Wu, 2005). The present study also demonstrated the *survivin-deltaEx3* gene expression to correlate with the nuclear pan-survivin expression. As a result of the above findings, the nuclear expression of pan-survivin could be the representation of both wild-type survivin and survivin-deltaEx3, both of which are associated with tumour proliferation. In contrast, the cytoplasmic expression of pan-survivin might indicate the presence of wild-type survivin and survivin-2B (Mahotka *et al*, 2002b). In fact, the present study revealed that the cytoplasmic pan-survivin expression was not associated with the apoptotic index. This result might be partly because cytoplasmic survivin-2B can inhibit the antiapoptotic function of wild-type survivin. We performed additional immunohistochemistry to clearly identify the subcellular localisation of survivin-deltaEx3 using a commercially available specific antibody against survivin-deltaEx3; however, we were not successful (data not shown).

As a result, the present study demonstrated the wild-type *survivin* gene expression to be associated with not only apoptotic inhibition but also tumour proliferation. In contrast, the *survivin deltaEx3* gene expression significantly correlated with the nuclear expression of pan-survivin and the Ki-67 proliferation index. However, the *survivin 2B* gene expression did not correlate with tumour proliferation or tumour apoptosis. Considering these results, the combined evaluations of wild-type *survivin* and *survivin-deltaEx3* gene expression may thus be a useful strategy for evaluating NSCLCs. The RNA inhibition of *survivin* can induce apoptosis in human tumour cell lines (Ambrosini *et al*, 1998; Olie *et al*, 2000). Therefore, the RNA inhibition designed against common regions of both the wild-type *survivin* and the *survivin-deltaEx3* could be a more effective strategy for NSCLCs not only to improve the efficacy of chemo-radio therapy but also to suppress tumour cell proliferation (Li *et al*, 1998; Lu *et al*, 2004).

## Figures and Tables

**Table 1:** Gene expression of wild-type *survivin*, *survivin-2B*, and *survivin-deltaEx3* in 134 NSCLCs according to clinical characteristics

		**Wild-type *survivin***		***Survivin-2B***		***Survivin-deltaEx3***	
**Variables**	***n***	**Standardised gene expression ratio**	***P*-value**	**Standardised gene expression ratio**	***P*-value**	**Standardised gene expression ratio**	***P*-value**
*Tumour status*							
T1	46	1.407±0.786	0.3145	1.472±1.094	0.6335	0.922±0.614	0.3219
T2	48	1.475±0.698		0.985±0.515		0.884±0.526	
T3	22	1.817±0.885		1.345±0.747		1.189±0.720	
T4	18	1.646±0.905		1.144±0.681		1.063±0.638	
							
*Nodal status*							
N0	86	1.459±0.835	0.1613	1.265±0.748	0.5628	0.902±0.564	0.0781
N1, N2, N3	48	1.660±0.707		1.175±0.914		1.096±0.673	
							
*Pathological stage*							
Stage I	60	1.385±0.819	0.0558	1.281±0.796	0.5626	0.833±0.512	0.0174
Stage II, III	74	1.649±0.759		1.194±0.860		1.084±0.661	
							
*Differentiation*							
Well	34	1.288±0.712	0.0383	1.318±0.832	0.4964	0.743±0.526	0.0110
Moderately or poorly	100	1.614±0.807		1.203±0.832		1.049±0.619	
							
*Histology*							
Adenocarcinoma	66	1.362±0.664	0.0181^a^	1.192±0.736	0.5191^a^	0.854±0.587	0.0377^a^
Squamous cell carcinoma	65	1.690±0.892		1.285±0.923		1.075±0.616	
Large cell carcinoma	3	1.780±0.570		0.850±0.507		1.321±0.620	
							
Total	134	1.531±0.795		1.233±0.830		0.971±0.610	

Abbreviation: NSCLCs=non-small cell lung cancers.

a Squamous cell carcinoma *vs* adenocarcinoma.

**Table 2:** Biological behaviours in relation to gene expressions of survivin variants and survivin protein expressions

	***n***	**Ki-67 proliferation index**		**Apoptotic index**		**Growth index**	
*Wild-type survivin*							
Positive	72	57.4±22.0	*P*<0.0001	18.1±5.6	*P*<0.0001	3.47±1.68	*P*<0.0001
Negative	62	36.2±28.5		23.1±7.2		1.66±1.37	
							
*Survivin-2B*							
Positive	72	50.0±26.4	*P*=0.1776	19.8±6.9	*P*=0.3399	2.86±1.76	*P*=0.0583
Negative	62	43.4±28.2		21.0±6.5		2.28±1.64	
							
*Survivin-deltaEx3*							
Positive	101	53.4±25.5	*P*<0.0001	20.0±6.7	*P*=0.1872	3.01±1.76	*P*<0.0001
Negative	33	29.8±25.0		21.8±7.1		1.48±1.33	
							
*Nuclear survivin*							
Positive	88	52.4±26.5	*P*=0.0047	19.9±7.0	*P*=0.2662	3.02±1.88	*P*=0.0004
Negative	46	38.5±26.6		21.3±6.5		1.90±1.33	
							
*Cytoplasmic survivin*							
Positive	98	48.3±26.9	*P*=0.6075	20.1±6.7	*P*=0.4464	2.70±1.81	*P*=0.4805
Negative	36	45.6±28.7		21.1±7.2		2.45±1.74	
